# Depth-Sensing Indentation Creep Behavior of Nanostructured Thermal Barrier Coatings from As-Synthesized t’-8YSZ Feedstocks

**DOI:** 10.3390/nano10010038

**Published:** 2019-12-23

**Authors:** Feifei Zhou, Min Liu, Yaming Wang, You Wang, Chunming Deng

**Affiliations:** 1 Department of Materials Science, School of Materials Science and Engineering, Harbin Institute of Technology, Harbin 150001, China; snowy_hit@163.com (F.Z.); wangyaming@hit.edu.cn (Y.W.); 2 National Engineering Laboratory for Modern Materials Surface Engineering Technology & The Key Lab of Guangdong for Modern Surface Engineering Technology, Guangdong Institute of New Materials, Guangzhou 510651, China; liumin_gz@163.net

**Keywords:** nanostructured 8YSZ coatings, indentation creep behavior, strain rate sensitivity, t’ feedstocks, atmospheric plasma spraying

## Abstract

Nano-indentation is a popular method to characterize the micromechanical properties of nanostructured 8YSZ coatings. However, little research has focused on the creep behavior of nano-indentation and only the elastic modulus and nanohardness have been analyzed. Herein, for the first time, the nano-indentation creep behavior of plasma-sprayed nanostructured 8YSZ coatings using as-prepared nanostructured non-transformable tetragonal (t’) feedstocks was investigated. The indentation creep behavior can be well characterized by the power-law equation and the strain rate sensitivity has been calculated in light of the equation. The strain rate sensitivity was sensitive to the load and the reasons were analyzed in detail. The current results can further guide and design thermal barrier coatings from the point of indentation creep property.

## 1. Introduction

Thermal barrier coatings (TBCs), whose significance goes without saying, are as important to hot end components of land-based gas turbine and aircraft engines as clothes are to human beings. TBCs are composed of multifunctional multilayers and multimaterials including the bond-coat and top-coat. The bond-coat is mainly comprised of a PtNiAl or MCrAlY (M = Ni or/and Co) composition and the state-of-the-art top-coat material is primarily ZrO_2_ stabilized by 6–8 wt.% Y_2_O_3_ (8YSZ) [1,2,3,4]. As for TBCs, their service environment is quite complex and harsh such as oxidation, corrosion, erosion, fatigue, and creep [5,6]. Although these factors bring great difficulties and challenges to the research and evaluation of coating performance, TBCs are still a research hotspot with the aim of making it “healthier” in service.

There are several routes to deposit coatings such as physical vapor deposition (PVD) [7], chemical vapor deposition (CVD) [8], and atomic layer deposition (ALD) [9]. In terms of thermal barrier coatings, the main deposition techniques are atmospheric plasma spraying (APS) and electron beam-physical vapor deposition (EB-PVD) [2]. APS is the most usual and extensive method to prepare TBCs [10,11]. Research and development of nanotechnology and nanomaterials have further promoted and expanded the development of APS [12]. The plasma-sprayed nanostructured 8YSZ coatings can be obtained using nanostructured powder feedstocks and it has been demonstrated that nanostructured 8YSZ coatings present more remarkable properties than microstructured counterparts [13,14,15,16,17,18,19,20]. Therefore, it is necessary and significant to develop nanostructured 8YSZ coatings.

Mechanical properties, embodying elastic modulus, hardness, fracture toughness and bonding strength, are extremely essential to ensure coatings with functionality, reliability, and durability during service [17,20,21,22]. In addition to tensile testing for bonding strength, nano-indentation is also required and useful for most measurements or calculation of these mechanical properties. Nano-indentation tests in references have mostly focused on the modulus of elasticity, nano-hardness, and calculation of the fracture toughness of coatings [23,24,25]. However, these references pay little attention to the creep behavior of nanostructured 8YSZ coatings. Concerning nanostructured 8YSZ thermal barrier coatings, the stress state and distribution have influences on the properties of coatings [1,6]. It has been reported that the creep property of 8YSZ coatings can determine the stress distribution [26]. Therefore, it is of great importance to study the creep property of nanostructured 8YSZ coatings.

Our previous work prepared and investigated high-performance non-transformable tetragonal (t’) 8YSZ feedstocks with nanostructure [27]. In this paper, we fabricated high-performance nanostructured 8YSZ coatings from t’ feedstocks using the APS method. The creep behavior of nanostructured 8YSZ coatings was studied by nano-indentation for the purpose of filling in this research gap.

## 2. Experimental 

The main thermal-sprayed powders were nanostructured t’-8YSZ feedstocks. The nanostructured 8YSZ coatings were gained by means of APS technology (Metco 9MC) using the feedstocks above-mentioned. The raw 8YSZ nanopowders—whose grain size was about 20 nm—were provided by Fujian Dilong Innovation Development Co. Ltd, Quanzhou, China. The feedstocks can be obtained by the nanopowder granulation method and the particle size distribution was d_10_ = 6.46 μm, d_50_ = 33.22 μm, d_90_ = 60.52 μm. The details regarding the fabrication of feedstocks can be seen in our previous publication [27]. Before depositing the 8YSZ coatings, NiCoCrAlYCe coatings were produced via high velocity oxygen fuel sprayed (JP-5000) onto a K417G superalloy substrate. The characterization of nanostructured t’-8YSZ coatings are reported in the relevant work [28]. The cross sectional morphologies of coatings were observed by scanning electron microscopy (SEM, Nova NanoSEM 430). The nano-indentation test (Nano Indenter G200, Agilent Technologies, USA) was implemented on the polished section of the coatings. The peak loads were 0.5 gf (4.8 mN), 5 gf (48 mN), and 50 gf (480 mN) with the holding time of 10 s. For each peak load, 18 indents were conducted at random for repeatability. 

## 3. Results and Discussion

**Figure 1:**
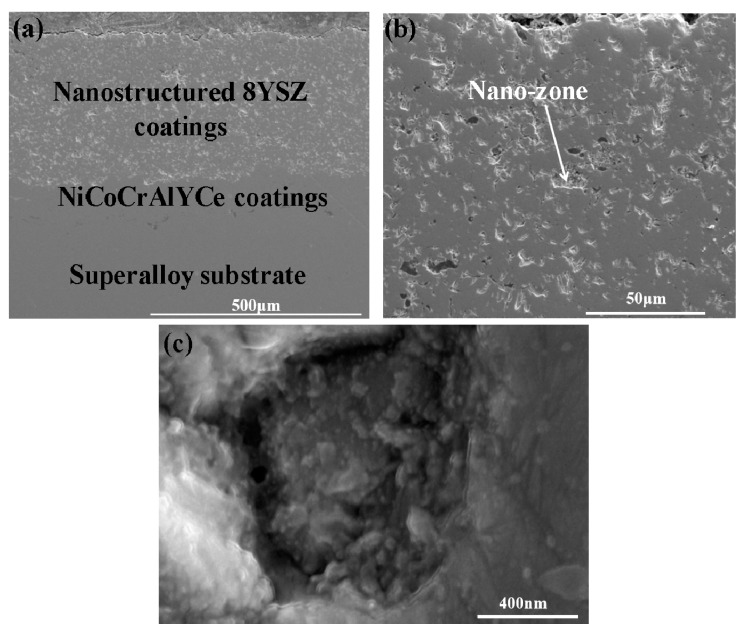
(**a**) Cross sectional SEM images of as-sprayed 8YSZ coatings, (**b**) magnified image of (**a**), and (**c**) magnified image of unmelted feedstocks in (**b**).

Figure 1 shows the cross sectional images of plasma-sprayed nanostructured 8YSZ coatings. The thickness of 8YSZ coatings was about 300 μm. It can be observed that the 8YSZ coatings consist of molten lamellae and non-molten nanostructured feedstocks. As shown in Figure 1c, the non-molten regions are also called nano-zones. This microstructure will influence the deformation behavior of the coatings. As for the nanostructured 8YSZ coatings, the mechanical properties such as elastic modulus and nanohardness in molten regions are higher than that in nano-zones and a detailed discussion can be observed in our previous work [28]. 

**Figure 2:**
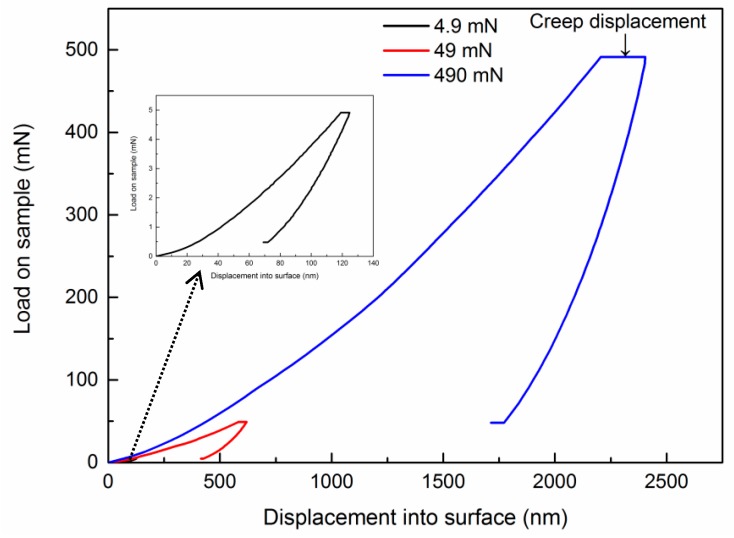
Load-displacement curves of nanostructured 8YSZ coatings at different loads with 10 s holding time.

Figure 2 illustrates the typical load-displacement curves at 4.9 mN, 49 mN, and 490 mN with 10 s holding time for nanostructured 8YSZ coatings. It is clear that the increase of creep displacement is more significant as the loads increase. According to the observation, it can be concluded that the creep behavior of 8YSZ coatings is sensitive to the applied load. When the load increases, the deformation zone of the coatings grows and the effect of microstructure defects such as pores and splat boundaries is pronounced.

The strain rate sensitivity is an important parameter for the creep behavior of nanostructured 8YSZ coatings. The equation describing the steady state creep can be given as follows [29,30]:

(1) $$\frac{1}{h}\frac{dh}{dt}=K\sigma^{\frac{1}{m}},$$

where *h* is the indentation depth; *t* is the time, *K* is the constant; *σ* is the flow stress; and *m* is the strain rate sensitivity. When the loads reach the peak values and the holding time starts, the flow stress can be expressed as [31]

(2) $$\sigma =C_{1}\frac{P_{\max}}{h^{2}},$$

where *C*_1_ is the constant and *P*_max_ is the peak load.

Substituting Equation (2) into Equation (1), Equation (3) can be obtained as follows:

(3) $$\frac{1}{h}\frac{dh}{dt}=K{\left(C_{1}\frac{P_{\max}}{h^{2}}\right)}^{\frac{1}{m}},$$

It can be supposed that the time at the beginning of creep is recorded as *t*_0_ and the indentation depth is *h*_0_ in the nano-indentation process. Therefore, the relationship between indentation depth (*h*) and time (*t*) can be gained by separating variables (*t*, *h*) and integrating from Equation (3). The integral Equation (4) is as follows:

(4) $${\int_{h_{0}}^{h}h}^{\frac{2}{m}-1}dh=\int_{t_{0}}^{t}KC_{1}{}^{\frac{1}{m}}P_{{}_{\max}}^{\frac{1}{m}}dt,$$

According to Equation (4), it can be derived as Equation (5).

(5) $$\frac{m}{2}h^{\frac{2}{m}}=KC_{1}P_{{}_{\max}}^{\frac{1}{m}}{t-KC}_{1}P_{\max}^{\frac{1}{m}}t_{0}+\frac{m}{2}h_{{}_{0}}^{\frac{2}{m}},$$

Equation (5) can be further taken as Equation (6).

(6) $$\frac{m}{2}h^{\frac{2}{m}}=C_{2}({t-t}_{c}),$$

where $C_{2}=KC_{1}P_{\max}^{\frac{1}{m}}$, $t_{c}=t_{0}-\frac{\frac{m}{2}h_{0}^{\frac{2}{m}}}{C_{2}}$

Based on Equation (6), the function of indentation depth (*h*) versus time (*t*) can be gained in Equation (7).

(7) $$h=A{\left({t-t}_{c}\right)}^{\frac{m}{2}},$$

where $A={\left(\frac{2C_{2}}{m}\right)}^{\frac{m}{2}}$. Equation (7) indicates that the strain rate sensitivity can be calculated by fitting the *h-t* data at holding time 

**Figure 3:**
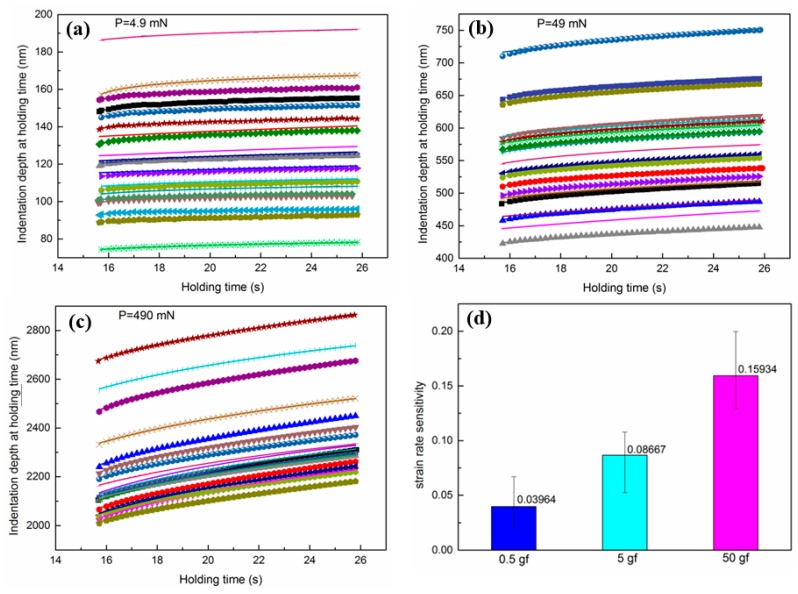
(**a**–**c**) Indentation depth as a function of holding time for nanostructured 8YSZ coatings at different loads using the fitting equation, (**d**) strain rate sensitivity of nanostructured 8YSZ coatings at different loads.

The data of indentation depth at holding time versus holding time at different loads and the fitting curves using Equation (7) are shown in Figure 3. It was easy to observe that the creep displacement increased significantly as the load increased. The fitting correlation coefficient (R^2^) was above 0.99, which means that Equation (7) fits very well. The results of calculated strain rate sensitivity are shown in Figure 3. As the load was 0.5 gf, the strain rate sensitivity of the nanostructured 8YSZ coatings was 0.03964. The strain rate sensitivity increased with the increase in load and was 0.08667 versus 5 gf and 0.15934 versus 50 gf. It seems that the strain rate sensitivity increased approximately twice with ten times the current load. The lower strain rate sensitivity indicates a greater resistance to the creep deformation [32]. When the load is lower, there will be smaller regions to be analyzed. The indentation size is about the same order as the single splat or a few splats in coatings and there is little effect from the defects in coatings on deformation. Therefore, the lower load leads to a lower strain rate sensitivity. This indentation size effect can also be observed in other materials during the indentation creep testing [31,33]. As the load increases, the deformation regions are larger. The coatings’ microstructure (including the splat boundaries and pores) will play a vital role in plastic deformation and hence a higher strain rate sensitivity can be seen. As for plasma-sprayed thermal barrier coatings, residual stress exists in coatings at room temperature [2,10]. The creep phenomenon can be observed for thermal barrier coatings under tensile stress or compressive stress at room temperature or relatively low temperature [34,35]. Therefore, the investigation regarding the creep property of 8YSZ thermal barrier coatings is very important [35,36]. We can design and guide new thermal barrier coatings materials including nanostructure and doping by indentation creep properties at room temperature, according to Equation (6).

## 4. Conclusions

In the present study, the nano-indentation creep behavior of nanostructured 8YSZ coatings was investigated for the first time. It has been demonstrated that the equation $h=A{\left({t-t}_{c}\right)}^{\frac{m}{2}}$ can well characterize the indentation creep process of nanostructured 8YSZ coatings. Furthermore, the strain rate sensitivity of coatings can be calculated based on the above equation. The strain rate sensitivity of nanostructured 8YSZ coatings increases as the load increases. The present study can provide some enlightenment as to the creep behavior of other nanostructured thermal barrier coatings such as nanostructured rare-earth zirconate materials. By analyzing the indentation creep property, we can further design and select novel thermal barrier coatings.
